# Modulation of soleus muscle H-reflexes and ankle muscle co-contraction with surface compliance during unipedal balancing in young and older adults

**DOI:** 10.1007/s00221-020-05784-0

**Published:** 2020-04-07

**Authors:** Leila Alizadehsaravi, Sjoerd M. Bruijn, Huub Maas, Jaap H. van Dieën

**Affiliations:** Department of Human Movement Sciences, Faculty of Behavioural and Movement Sciences, Institute for Brain and Behaviour Amsterdam and Amsterdam Movement Sciences, Vrije Universiteit Amsterdam, Van der Boechorststraat 9, 1081 BT Amsterdam, The Netherlands

**Keywords:** Balance control, Postural control, Spinal excitability, H-reflex, Aging, Co-contraction

## Abstract

This study aimed to assess modulation of lower leg muscle reflex excitability and co-contraction during unipedal balancing on compliant surfaces in young and older adults. Twenty healthy adults (ten aged 18–30 years and ten aged 65–80 years) were recruited. Soleus muscle H-reflexes were elicited by electrical stimulation of the tibial nerve, while participants stood unipedally on a robot-controlled balance platform, simulating different levels of surface compliance. In addition, electromyographic data (EMG) of soleus (SOL), tibialis anterior (TA), and peroneus longus (PL) and full-body 3D kinematic data were collected. The mean absolute center of mass velocity was determined as a measure of balance performance. Soleus H-reflex data were analyzed in terms of the amplitude related to the M wave and the background EMG activity 100 ms prior to the stimulation. The relative duration of co-contraction was calculated for soleus and tibialis anterior, as well as for peroneus longus and tibialis anterior. Center of mass velocity was significantly higher in older adults compared to young adults ($$p<0.001)$$ and increased with increasing surface compliance in both groups ($$p<0.001)$$. The soleus H-reflex gain decreased with surface compliance in young adults $$(p= 0.003)$$, while co-contraction increased $${(p}_{\mathrm{S}\mathrm{O}\mathrm{L},\mathrm{T}\mathrm{A}}=0.003\ \mathrm{a}\mathrm{n}\mathrm{d}\ {p}_{\mathrm{P}\mathrm{L},\mathrm{T}\mathrm{A}}<0.001)$$. Older adults did not show such modulations, but showed overall lower H-reflex gains $$(p<0.001)$$ and higher co-contraction than young adults $${(p}_{\mathrm{S}\mathrm{O}\mathrm{L},\mathrm{T}\mathrm{A}}<0.001\ \mathrm{a}\mathrm{n}\mathrm{d}\ {p}_{\mathrm{P}\mathrm{L},\mathrm{T}\mathrm{A}}=0.002)$$. These results suggest an overall shift in balance control from the spinal level to supraspinal levels in older adults, which also occurred in young adults when balancing at more compliant surfaces.

**Electronic supplementary material:**

The online version of this article (10.1007/s00221-020-05784-0) contains supplementary material, which is available to authorized users.

## Introduction

In upright stance, balance is challenged by gravity and the relatively high position of the body center of mass (CoM) over a small base of support. This challenge increases with impairments in neuromuscular control resulting from age or disease (Pasma et al. 2015). But even for young, healthy individuals, maintenance of balance can become challenging when their base of support is reduced or when compliance of the surface which they are standing on is increased (Raymakers et al. 2005; Schut et al. 2017).

In balancing on a rigid surface, moments around the ankle joint instantaneously and proportionally change the position of the center of pressure and therewith cause moments that accelerate the body center of mass (Hof 2007). On a compliant surface, moments around the ankle joint change the center of pressure by moving or deforming the support surface. Consequently, the relation between the ankle moment and the center of mass acceleration is different than on a rigid surface, with changes in scaling of the effect of changes in ankle moment as well as in the temporal relation between the moment and the resulting center of mass acceleration. When standing on a compliant surface, also the relationship between sensory information from the calf muscles and the orientation of the body relative to the vertical changes. For example, with the body perfectly vertical, the ankle can still be in any orientation, as body orientation and ankle angle are decoupled. Consequently, ankle angle provides little-to-no information on body orientation. Balance control could potentially be adapted to such a challenge in various ways.

Considering the above, one would expect proprioceptive afference from sensors in the lower extremities to be less used when standing on a compliant surface compared to a rigid surface. In line with this, effects of calf muscle vibration, triggering muscle spindle afference, are less pronounced when standing on a compliant compared to a rigid surface (Ivanenko et al. 1997; Kiers et al. 2012). This effect could be accounted for by sensory reweighting (van Dieën et al. 2015) or supraspinal suppression of motoneuron excitability. Supporting the latter mechanism, long-term training on compliant surfaces does suppress H-reflexes (Taube et al. 2008; Keller et al. 2012), but it is not clear whether immediate modulation of H-reflexes to surface compliance occurs. Experiments using a reduced base of support show that indications of immediate modulations in reflex sensitivity, i.e., a negative correlation between postural demands (standing with wide or narrow base of support, prone or standing, and bipedal or unipedal stance) and H-reflex amplitudes have been reported (Koceja et al. 1995; Tokuno et al. 2009; Kawaishi and Domen 2016; Pinar et al. 2010; Kim et al. 2013). Koceja and Mynark (2000) revealed that down-modulation of the H-reflex was associated with greater postural stability, underlining the adaptive nature of this modulation. Increased postural demands also coincide with increased cortical activity (Papegaaij et al. 2016b). These findings suggest inhibition of peripheral (spinal) control mechanisms and an increased supraspinal contribution to balance control with increasing task difficulty (Papegaaij et al. 2014), and considering the above, this might apply specifically to increasing surface compliance. The ability to adapt balance control to surface conditions is a prerequisite to safely move through a variable environment.

Aging causes impairments of the balance control system due to degeneration of gray and white brain matter and peripheral nerves, decreased acuity of the sensory systems, and diminished muscular capacity (Cham et al. 2007; Papegaaij et al. 2014). Age-related reductions in H-reflex amplitudes (Koceja et al. 1995) and increased cortical engagement in motor control (Kahya et al. 2019) indicate an increased contribution of cortical relative to spinal inputs to balance control (Papegaaij et al. 2014) which may reflect a bigger postural challenge in this group. Presumably, older adults need more cortical control to cope with the same task in view of age-related changes in balance control mechanisms. Older adults are also known to display increased co-contraction in postural tasks (Iwamoto et al. 2017), which may be caused by inadequate inhibition of antagonistic muscles leading to increased joint stiffness, possibly resulting in an increased susceptibility to fall (Tucker et al. 2009). In contrast, increased co-contraction could be a compensatory strategy for impaired balance control (Kaplanski 2001), as it reduces delays in feedback control through pre-tensioning of muscle–tendon complexes (Oomen et al. 2015).

In addition to experiencing an overall increase in the challenge of controlling balance, older adults appear to be less able to adapt balance control to varying environmental conditions (Pasma et al. 2015). Young adults were shown to down-modulate the soleus H-reflex between prone and standing, while older adults showed no modulation (Koceja and Mynark 2000) or even up-modulation with postural demands (Koceja et al. 1995; Angulo-Kinzler et al. 1998).

The aim of this study was to investigate the effects of varying surface compliance in mediolateral direction on single-leg balance control by assessing modulation of spinal excitability and duration of co-contraction of lower leg muscles in older compared to young adults. To the best of our knowledge, this is the first study comparing immediate adaptation in mediolateral balance control to variations in surface compliance between young and older adults. We hypothesized that balance performance decreases with increasing surface compliance and that young adults show down-modulation of spinal reflexes with increasing surface compliance. In addition, we hypothesized that older adults show less modulation of spinal reflexes and more co-contraction than young adults.

## Methods

### Participants

Ten young [28.2 $\pm$ 1.3 years (mean $\pm \mathrm{S}\mathrm{D})$, two females, weight 70.4 $\pm$ 16.3 kg (mean $\pm \mathrm{S}\mathrm{D})$, height 176.2 $\pm$ 10.0 cm (mean $\pm \mathrm{S}\mathrm{D})$] and ten older [71.4 $\pm$ 3.9 years (mean $\pm \mathrm{S}\mathrm{D})$, three females, weight 79.0 $\pm$ 11.9 kg (mean $\pm \mathrm{S}\mathrm{D})$, height 173.3 $\pm$ 10.0 cm (mean $\pm \mathrm{S}\mathrm{D})$] healthy volunteers participated in this study. All younger participants were recruited through flyers distributed at Faculty of Behavioral and Movement Sciences, VU Amsterdam. All older participants were recruited through a list of older adults who previously participated in the research at our faculty, flyers, and information sharing meetings at European science night. Individuals with peripheral neuropathy, self-reported orthostatic complaints, severe visual or hearing impairments and use of medication that may negatively affect balance were excluded. All participants provided written informed consent before participation and the procedures were approved by the ethical review board of the Faculty of Behavioral & Movement Sciences, VU Amsterdam (VCWE-2018-038).

### Instruments and data recordings

**Fig. 1:**
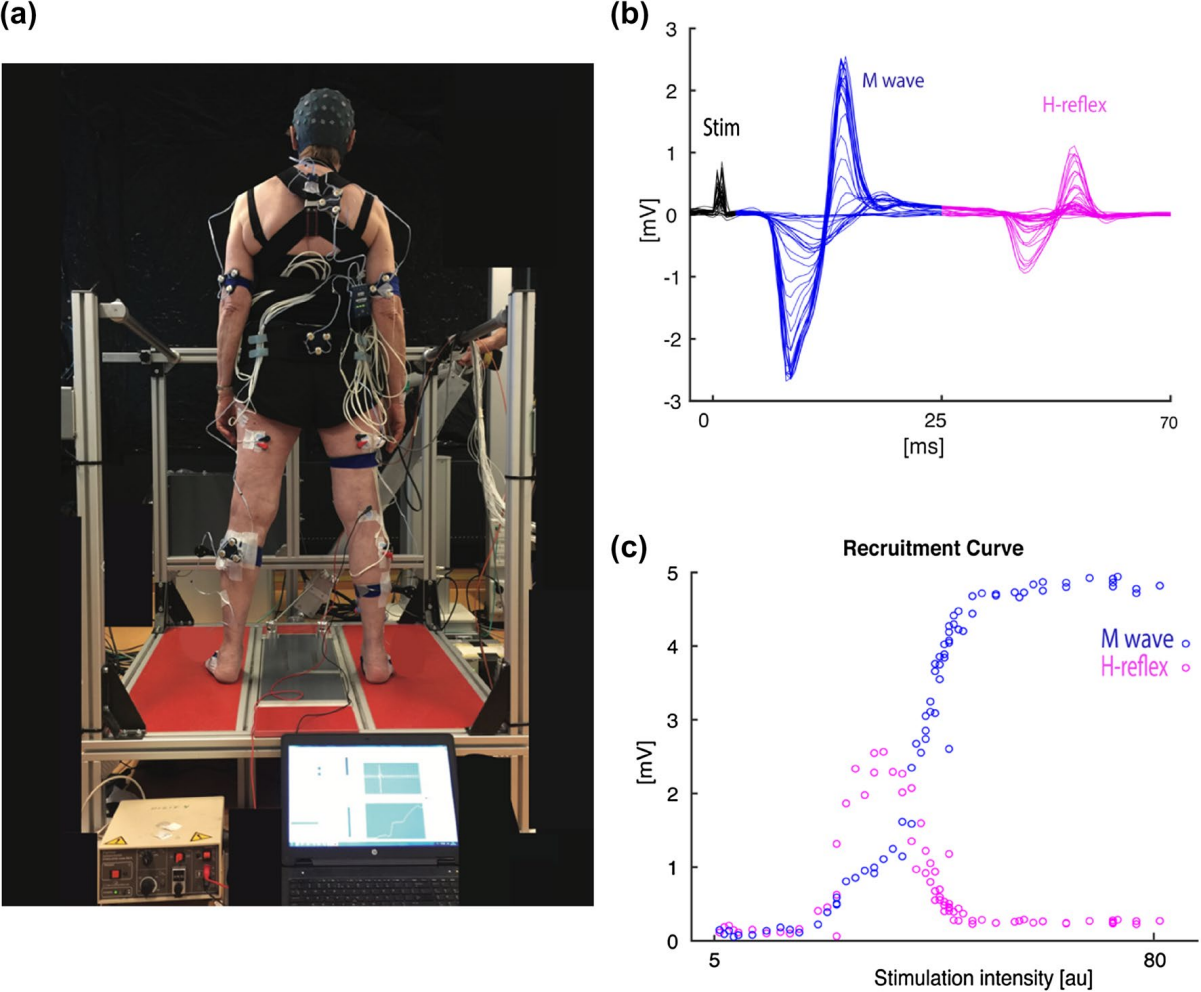
**a** Experimental setup, showing a participant in bipedal stance, receiving electrical stimulation to establish the recruitment curve. **b** Time series of the EMG response of the soleus muscle to the stimulation, showing traces at different stimulus intensities, each with a stimulus artifact (Stim), an M wave, and an H-reflex. **c** Recruitment curves, showing peak-to-peak values of M waves and H-reflexes as a function of stimulus intensity

Surface conditions were induced using a custom-made robot-controlled (HapticMaster, Motekforce Link Amsterdam, The Netherlands) platform with a footplate rotating in the frontal plane (Fig. 1a). Rotational stiffness of the footplate and damping was tunable and controlled with a simulated spring. Maximal rotation of the footplate was ± 17.5°.

Full-body kinematics were acquired with one Optotrak camera array (Northern Digital, Waterloo, ON, Canada) at 50 samples/s. Six Optotrak LED marker clusters were placed on the posterior surface of the thorax, pelvis, arms, and calves. The markers were tracked by the camera and anatomical landmarks were digitized in an upright posture, using a pointing probe with six markers.

Electromyographic (EMG) data were collected at 2048 samples/s by a TMSi Refa 128-channel amplifier (TMSi, Twente, The Netherlands) data acquisition system. EMG data of the soleus, peroneus longus, and tibialis anterior muscles of the stance leg were collected using bipolar, disposable adhesive surface electrodes (Ag/AgCl EMG electrodes, Ambu blue sensor N, Ambu, Ballerup, Denmark). Electrode sites were prepared by shaving the area when needed. To reduce the impedance at the skin–electrode interface, the electrode sites were cleaned with 70% isopropyl alcohol swabs. The electrode placement was chosen according to the Surface EMG for Non-Invasive Assessment of Muscles (SENIAM) recommendations (Hermens et al. 2000). A reference electrode was placed on the lateral malleolus of the stance leg.

H-reflexes were elicited using an electrical stimulator delivering 1-ms square-wave pulses (Digitimer, DS7A UK). A large rectangular anode, roughly 6 cm × 9 cm, constructed of aluminum foil and conducting gel was fixed on the patella (Zehr 2002). The cathode for unipolar stimulation was placed over the tibial nerve in the popliteal fossa to elicit an H-reflex in the soleus muscle. The optimal stimulation location was determined in each subject by probing the popliteal fossa with a custom-made probe for the location where the largest soleus H-reflex amplitude appeared ~ 25 ms after the stimulation.

### Experimental procedures

Explanation and familiarization of the peripheral nerve stimulation procedure and postural conditions were provided prior to testing. To control for potential attentional and anticipatory influences on spinal reflex excitability, consistent lighting and minimal auditory input were ensured throughout the experiment. First, soleus H-reflex threshold intensity was determined using percutaneous electrical stimulation of the posterior tibial nerve during quiet, bipedal stance, and then, stimulus intensity was progressively increased, with a minimum 4 s interval, to determine the maximum H-reflex response (Hmax) and maximal M wave (Mmax) (Fig. 1b, c) (Gruber et al. 2007). During this phase, participants were instructed to visually focus on a target while standing on both legs with their hands on their hips. Although soleus is not the most dominant muscle contributing to mediolateral balance control, it has a critical role to maintain the dynamic balancing in the frontal plane (Neptune and McGowan 2016; Cohen et al. 2018) and also soleus activation is crucial to keep the body upright, while the other muscles are stabilizing the body in the frontal plane (Sozzi et al. 2013). Moreover, H-reflexes can be reliably elicited in the soleus (Capaday and Stein 1986); therefore, we selected this muscle for studying H-reflexes.

Subsequently, ten H-reflexes were elicited using the Hmax constant current stimulus, during unipedal stance on the balance platform at various levels of surface compliance, with three repetitions. It should be noted that during the dynamic balancing, there could be changes in electrode location with respect to the nerve. Because the recruitment curve of the H-reflex is least steep around Hmax, H-reflexes are less likely affected by such changes. Thus, using the maximum H-reflex, we attempted to reduce errors caused by movements.

**Fig. 2:**
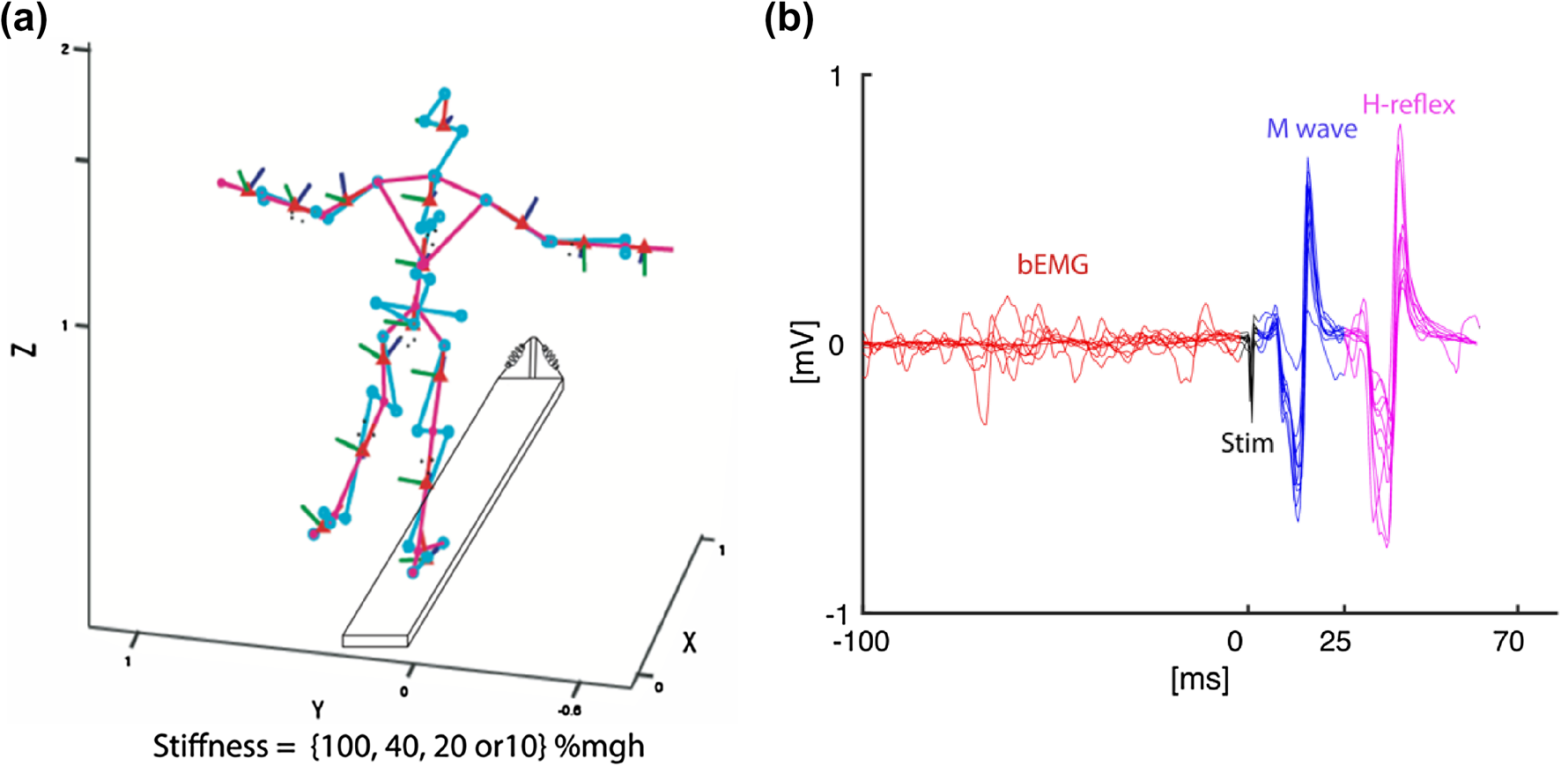
**a** Kinematic model used to assess balance performance during the unipedal balance task. **b** Epoched EMG data synchronized to stimulation artifacts (Stim) obtained during a balance task, showing background EMG 100 ms prior to the stimulation (bEMG), M wave, and H-reflex

During the testing phase, participants were instructed to focus on a target in front of them, with their arms slightly abducted and their hands above the handrails of the platform, while trying to stabilize the platform in a horizontal position (Fig. 2a). Participants were instructed to avoid flexing their stance leg knee during the task. A 10–15 s rest was provided between stimuli to avoid influences of post-activation depression. Thus, in total, 12 balance trials were performed, of 140 s each, grouped into three identical blocks (randomized per subject), each consisting of four varying levels of surface compliance (rotational stiffness set at 100%, 40%, 20%, and 10% of body weight multiplied by CoM height) randomized within blocks. Additionally, four trials of 60 s without stimulation at each compliance level were performed, to assess balance performance without stimulation. Participants were given a break of 2 min between trials, or as long as needed to avoid any effects of fatigue.

### Data analysis and statistics

#### Measures of balance performance

**Fig. 3:**
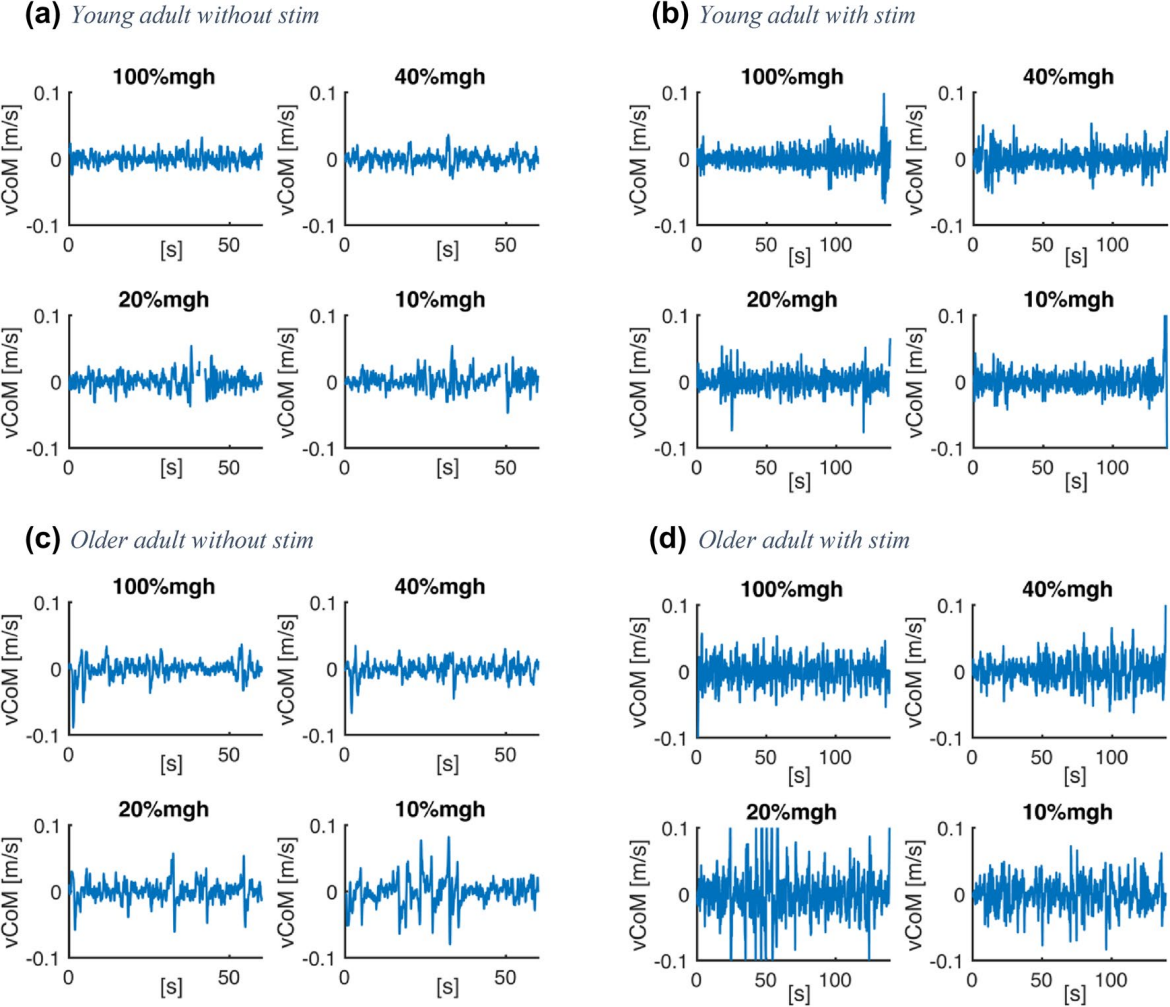
Time series of CoM velocity in one young and one older participant as a function of surface compliance in trials with and without stimulation at four levels of surface compliance (rotational stiffness set at 100%, 40%, 20%, and 10% of body weight multiplied by CoM height): **a** young adult without peripheral nerve stimulation, **b** young adult with peripheral nerve stimulation, **c** older adult without peripheral nerve stimulation, and **d** older adult with peripheral nerve stimulation. In both with/without peripheral nerve stimulation conditions, older adults display higher CoM velocity than younger adults, and both older and younger adults show increased CoM velocity with surface compliance

Missing samples of marker coordinates were interpolated by cubic spline interpolation, and marker coordinates were low-pass filtered with a cut-off frequency of 5 Hz. The trajectories of the segments were calculated using a 3D linked segmented model (Fig. 2a; Kingma et al. 1996) based on the coordinates of markers and anatomical landmarks. The total body CoM position and velocity (derivative of CoM position with respect to time, vCoM) were calculated (van Dieën et al. 2015). The arm segments were excluded, in view of invisibility of markers at time that participants moved their arms in front of their bodies. Supplementary material 1 shows that our analysis with arms included yielded similar results. The mean absolute vCoM, equivalent to the total excursion of the CoM divided by trial length, was used as a measure of balance performance (Raymakers et al. 2005; Fig. 3). This was done both for trials during which stimulation took place, and for trials without stimulation. In trials with stimulation, the results were averaged over repeated trials at an identical surface compliance.

#### Measures of soleus H-reflex excitability

All EMG signals were high-pass filtered at 10 Hz (second-order bi-directional Butterworth filter) to remove movement artifacts. The amplitude of the M wave was determined as the peak-to-peak amplitude of the EMG from 0 to 25 ms after the stimulus artifact, and the H-reflex amplitude was calculated as the peak-to-peak amplitude from 25 to 70 ms after the stimulus artifact. The amplitude of the background EMG (bEMG) was determined as the average rectified EMG signal over 100 ms before the stimulation (Fig. 2b). H/M ratio, the ratio of H-reflex amplitude and corresponding M wave amplitude, and the H-reflex gain [defined as the ratio of H-reflex amplitude divided by the bEMG (Hoffman and Koceja 1995)] were calculated. Applying bEMG normalization, we aimed to remove the effect of pre-existing motoneuron excitation (Verrier 1985; Bove et al. 2006). The amplitude of the H-reflex is expected to linearly increase with the level of muscle activation (Funase and Miles 1999; Taube et al. 2019). Therefore, the H-reflex gain was considered the main outcome. Nevertheless, changes in the H-reflex gain may be the result of changes in the background EMG. Therefore, we analyzed SOL bEMG averaged over the entire trial and normalized to bEMG during bipedal standing.

To check for consistency with the previous work (Pinar et al. 2010; Kim et al. 2013), we compared H-reflex amplitudes between unipedal and bipedal stance. Then, we calculated the above parameters for each surface compliance condition in unipedal stance. Note that during all unipedal stance trials, the H-reflex was elicited at the stimulus intensity of Hmax in bipedal stance.

#### Measure of co-contraction

**Fig. 4:**
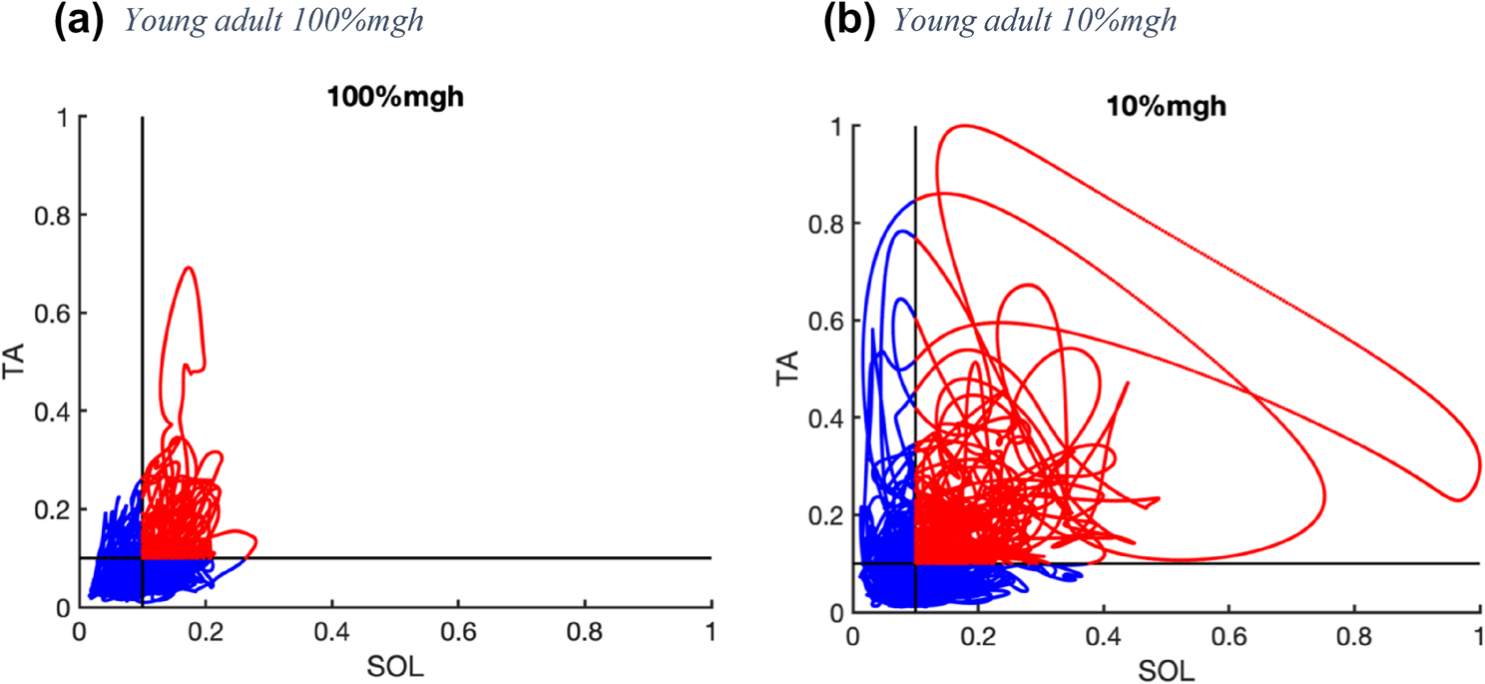
Co-contraction; results are displayed as scatter plots of tibialis anterior (TA, *y*-axis) and soleus (SOL, *x*-axis) activity of one young participant for two surface compliances, 100% and 10% of the product of body mass, gravity, and the height of the CoM (mgh). All data points were normalized to the maximum activity over all trials. Data points in red indicate co-contraction (both muscles active over 10% of maximum). Data points in blue indicate no co-contraction: **a** SOL TA in a young adult at 100%mgh; **b** SOL TA in a young adult at 10%mgh

All EMG signals were first high-pass filtered at 10 Hz (second-order bi-directional Butterworth filter) to remove movement artifacts, then rectified, and low-pass filtered at 5 Hz (second-order Butterworth). We assessed the duration of co-contraction of soleus and tibialis anterior as well as peroneus longus and tibialis anterior antagonistic muscle pairs. To this end, we determined the percentage of data points during the balance tasks without stimulation of the tibial nerve during which both muscles in a pair exceeded 10% of their maximum activation over all trials (Fig. 4).

#### Statistical analysis

All data are reported as means $\pm$ SDs. For all independent variables (absolute mean of vCoM, H-reflex excitability, SOL bEMG, and co-contraction), we evaluated the effect of surface compliance and age using a two-way mixed model ANOVA with age (young, old) as between-subjects factor and surface compliance (high-to-low stiffness, four levels) as within-subjects factor. In case of interactions, post hoc one-way ANOVAs were performed to test for effects of surface compliance within groups.

To verify that our H-reflex protocol replicated the previous studies (Pinar et al. 2010; Kim et al. 2013), we additionally performed a two-way mixed model ANOVA with factors age (young, old) and stance condition (bipedal-to-unipedal). This analysis was also done for SOL bEMG during bipedal and unipedal stance. All analyses were done in JASP version 0.9.2 (University of Amsterdam, The Netherlands), and *p* < 0.05 was considered significant.

## Results

### Balance performance

**Fig. 5:**
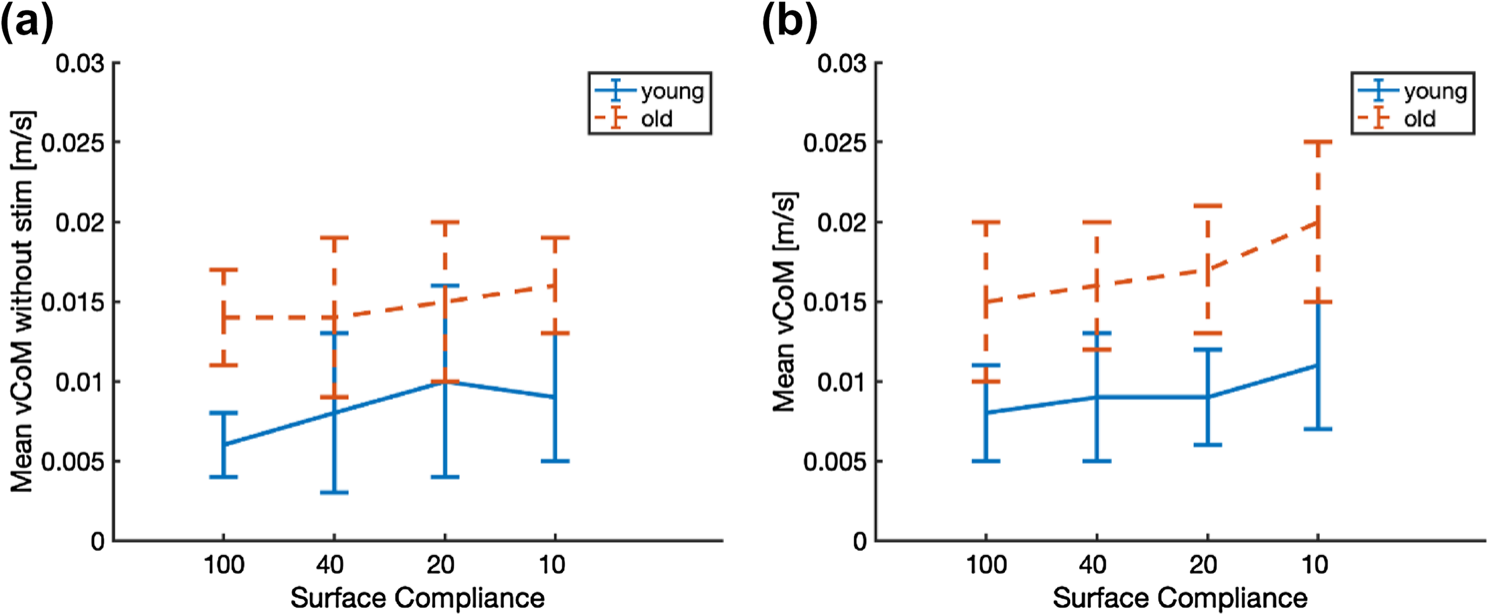
CoM velocity was higher in older than younger adults and increased with surface compliance. Displayed are group averaged values of the mean absolute CoM velocity as a function of surface compliance in trials **a** without stimulation of the tibial nerve (*n*_old_ = 9, *n*_young_ = 9) and **b** with stimulation of the tibial nerve (*n*_old_ = 10, *n*_young_ = 8) in young and older adults. Error bars represent standard deviations. Stiffness of the surface is expressed in % of subject weight multiplied by the height of the CoM

CoM velocity in the trials without and with tibial nerve stimulation was smaller in young than older adults (*F*_(1,16)_ = 12.724, *p* = 0.003; *F*_(1,16)_ = 20.013, *p* < 0.001 respectively) and increased with increasing surface compliance (*F*_(3,48)_ = 3.540, *p* = 0.021; *F*_(3,48)_ = 10.772, *p* < 0.001 respectively) (for typical examples, see Fig. 3). No significant interaction effect of surface compliance and age group was observed (*F*_(3,48)_ = 0.928, *p* = 0.435; *F*_(3,48)_ = 0.696, *p* = 0.599 respectively). Thus, the compliant surface increased the balance challenge with decreasing stiffness, and the challenge was always greater in older than in young adults (see Fig. 5a, b).

### Soleus H-reflex excitability

**Fig. 6:**
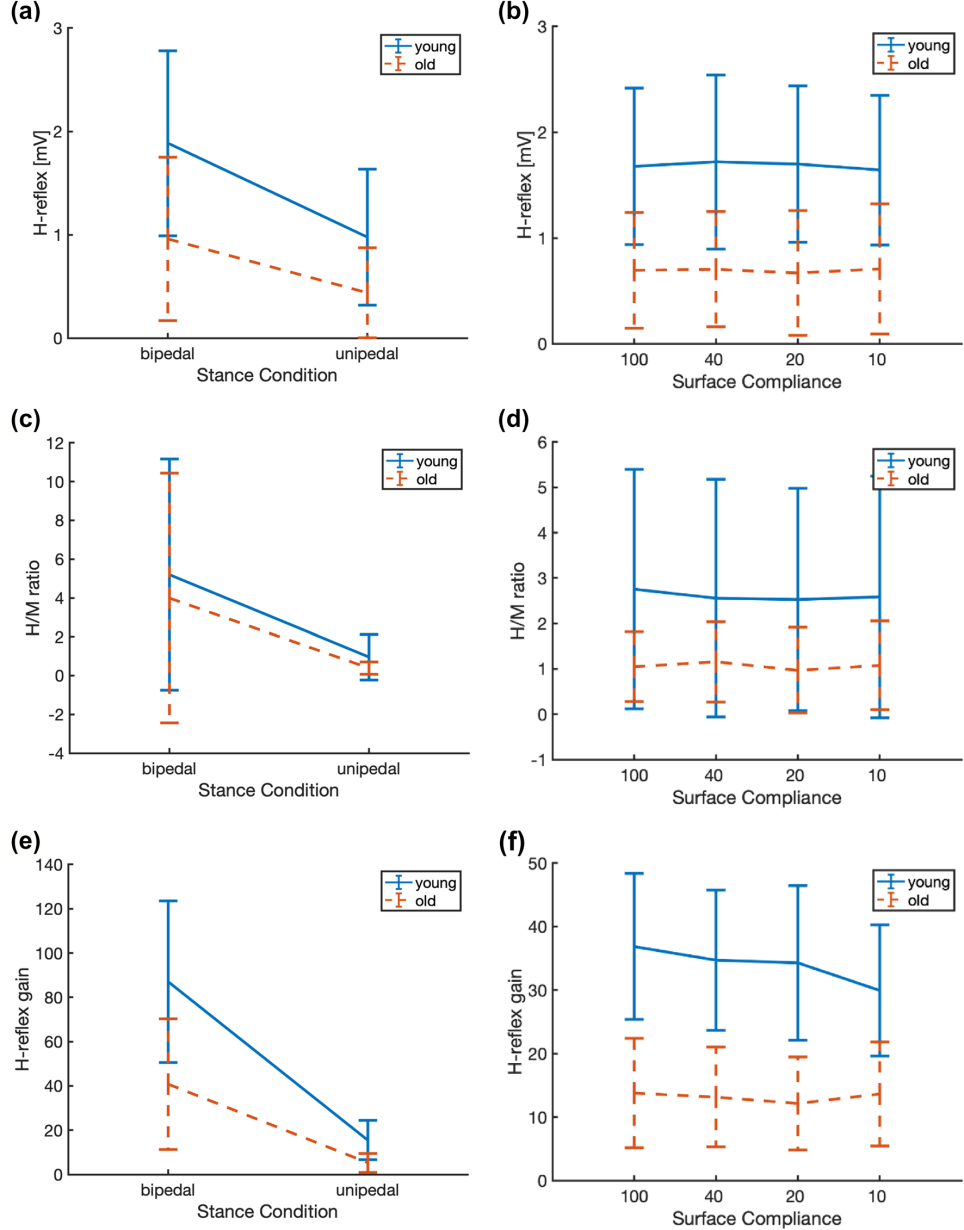
H-reflex amplitude, H/M ratio, and H-reflex gain as a function of stance condition (*n*_old_ = 10, *n*_young_ = 10) in **a**, **c,** and **e,** respectively and as a function of surface compliance (*n*_old_ = 10, *n*_young_ = 9) in **b**, **d,** and **f,** respectively, in young and older participants. Note that decreasing stiffness from left to right on the *x*-axis equates increasing surface compliance. H-reflex gain was higher in younger than older adults and decreased with stance condition. H-reflex gain is down-modulated with surface compliance only in young adults

A typical example of the H-reflex responses is shown in Fig. 2b. The results of H-reflex amplitude, H/M ratio, and H-reflex gain modulation due to surface compliance (see Fig. 6b, d and f) and stance condition (see Fig. 6a, c and e) are presented in Tables 1 and 2, respectively.

**Table 1:** Statistical results of the comparison of H, H/M, and H-reflex gain between age groups and surface conditions, bold numbers indicate a significant effect

Reflex unipedal	*df*1	*df*2	H		H/M		H-reflex gain	
			*F*	*p*	*F*	*p*	*F*	*p*
Surface compliance	3	51	0.221	0.881	0.659	0.581	4.679	**0.006**
Age	1	17	10.56	**0.005**	2.926	0.105	22.42	**< .001**
Surface compliance** × **age	3	51	0.420	0.074	0.639	0.593	4.895	**0.005**

**Table 2:** Statistical results of the comparison of H, H/M, and H-reflex gain between age groups and standing conditions, bold numbers indicate a significant effect

Reflex bipedal-to-unipedal	*df*1	*df*2	H		H/M		H-reflex gain	
			*F*	*p*	*F*	*p*	*F*	*p*
Stance condition	1	18	26.45	**< 0.001**	8.220	**0.010**	57.79	**< 0.001**
Age	1	18	6.435	**0.021**	0.386	0.542	12.16	**0.003**
Stance condition** × **age	1	18	1.922	0.183	0.056	0.815	6.505	**0.020**

There was no significant effect of surface compliance nor an interaction of surface compliance and age group, on H-reflex amplitude (*F*(3,51) = 0.221, *p* = 0.881; *F*(3,51) = 0.420, *p* = 0.074, respectively, see Fig. 6b). However, there was a significant effect of age group on H-reflex amplitude, indicating higher H-reflex amplitudes in young than older adults (*F*(1,17) = 10.56, *p* = 0.005, see Fig. 6b). There was no significant effect of surface compliance, age group, nor an interaction of surface compliance and age group on H/M ratio (*F*(3,51) = 0.659, *p* = 0.581; *F*(1,17) = 2.926, *p* = 0.105; *F*(3,51) = 0.639, *p* = 0.593 respectively, see Fig. 6d). Significant effects of surface compliance, age group, and an interaction of surface compliance and age group on the H-reflex gains were found (*F*(3,51) = 4.679, *p* = 0.006; *F*(1,17) = 22.42, *p* < 0.001; *F*(3,51) = 4.895, *p* = 0.005 respectively, see Fig. 6f) and post hoc testing indicated that there was no significant effect of surface compliance on H-reflex gain in the older participants (*F*(3,27) = 1.738, *p* = 0.186). This is in contrast to the young adults who showed smaller H-reflex gains on more compliant surfaces (*F*(3,27) = 5.929, *p* = 0.003, see Fig. 6f). In summary, our hypothesis that reflex sensitivity would be down-modulated with increasing surface compliance in young but not in older adults was supported by the H-reflex gains. In addition, note that no significant M-wave variation was observed with different compliance (*F*(3,51) = 1. 153, *p* = 0.337).

There were significant effects of stance condition and age group on H-reflex amplitudes, indicating smaller H-reflex amplitude in unipedal compared to bipedal stance and smaller H-reflex amplitude in older compared to young adults (*F*(1,18) = 26.45, *p* < 0.001, *F*(1,18) = 6.435, *p* = 0.021, respectively, see Fig. 6a). There was no significant interaction effect observed (*F*(1,18) = 1.922, *p* = 0.183). There was a significant effect of stance condition on H/M ratio indicating smaller H/M ratio in unipedal compared to bipedal stance (*F*(1,18) = 8.22, *p* = 0.010, see Fig. 6c), but no significant effect of age group nor an interaction of age group and stance condition on H/M ratio (*F*(1,18) = 0.386, *p* = 0.542, *F*(1,18) = 0.056, *p* = 0.815 respectively). We found smaller H-reflex gains in unipedal stance than in bipedal stance in both age groups and smaller H-reflex gains in older than young adults [(*F*(1,18) = 57.79, *p* < 0.001); *F*(1,18) = 12.16, *p* = 0.003, respectively, see Fig. 6e]. However, a significant interaction of stance condition and age was found *F*(1,18) = 6.505, *p* = 0.020) and post hoc tests revealed a stronger effect of stance condition in the young participants (*F*(1,9) = 41.582, *p* < 0.001) than in the older participants (*F*(1,9) = 16.774, *p* = 0.003) (Table 2). Overall, these results indicate reduced H-reflex sensitivity in unipedal compared to bipedal stance and decreased sensitivity in older compared to young adults, in line with previously reported findings.

**Fig. 7:**
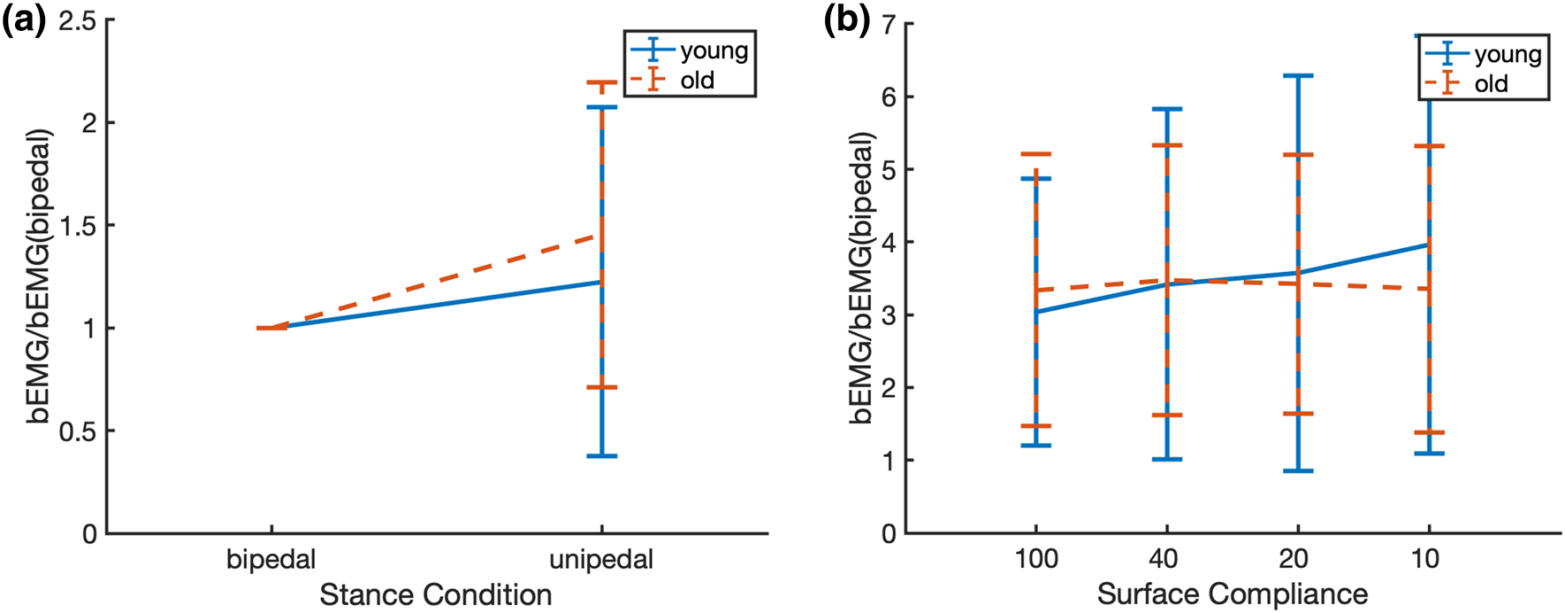
Normalized bEMG of SOL in young and older adults for both stance conditions in **a** and for the four surface compliance conditions in **b**

Decreased H-reflex gains with age, unipedal stance, and increased surface compliance could be due to increased bEMG. To test this, we compared normalized SOL bEMG between age groups and stance conditions. There were no significant age and stance effects, nor an interaction effect of age and stance condition on normalized bEMG (*F*(1,18) = 0.408, *p* = 0.531, *F*(1,18) = 3.603, *p* = 0.074, *F*(1,18) = 0.408, *p* = 0.531, respectively, Fig. 7a). For surface conditions, we found no age or surface compliance effects, nor an interaction effect of age and surface compliance on normalized bEMG (*F*(1,17) = 0.010, *p* = 0.921, *F*(3,51) = 2.703, *p* = 0.055, *F*(3,51) = 2.632, *p* = 0.06, respectively; Fig. 7b). It should be noted that effects of stance condition and surface were borderline significant.

### Co-contraction

**Fig. 8:**
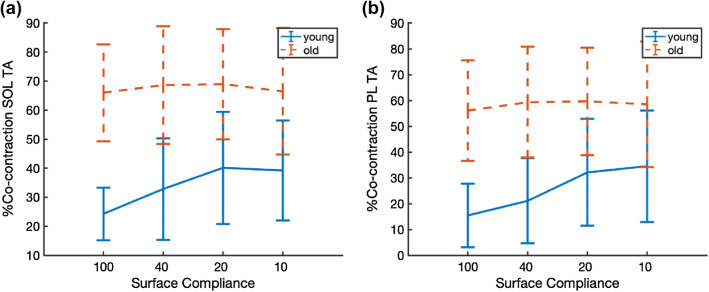
Co-contraction was not modulated with surface compliance in older adults but higher than younger adults. While in younger adults, co-contraction increased with surface compliance. Displayed are group relative duration of co-contraction of **a** soleus and tibialis anterior and **b** peroneus longus and tibialis anterior as a function of surface compliance in trials without peripheral nerve stimulation in young and older adults (*n*_old_ = 10, *n*_young_ = 10). Note that decreasing stiffness from left to right on the *x*-axis equates increasing surface compliance

The duration of co-contraction for both muscle pairs on average was higher in older adults and increased by surface compliance, but only in young adults. The duration of co-contraction of SOL, TA and PL, TA were higher in older compared to young adults (*F*_(1,17)_ = 18.37, *p* < 0.001; *F*_(1,17)_ = 14.22, *p* = 0.002, respectively, see Fig. 8a, b) and increased by surface compliance (*F*_(3,51)_ = 6.069, *p* = 0.001; *F*_(3,51)_ = 7.544, *p* < 0.001, respectively, see Fig. 8a, b). Significant interactions of age group and surface compliance were found for the duration of co-contraction of SOL, TA and PL, TA, and post hoc testing indicated an effect of surface compliance in young participants (*F*_(3,24)_ = 5.725, *p* = 0.004; *F*_(3,24)_ = 9.537, *p* < 0.001, respectively), but not in older participants (*F*_(3,27)_ = 0.909, *p* = 0.449; *F*_(3,27)_ = 0.471, *p* = 0.705, respectively, see Fig. 8a and b).

## Discussion

We investigated differences in balance control between young and older adults on surfaces with varying compliance. In line with our hypothesis, we found that (1) balance performance decreased with increasing surface compliance in both young and older adults, (2) older adults showed poorer balance performance than young adults, (3) young adults showed down-modulation of H-reflex gains, although absolute H-reflex amplitudes and H/M ratios were not affected, and an increase in co-contraction with increasing surface compliance, and (4) older adults showed no modulation of H-reflex gains or co-contraction with increasing surface compliance, but lower H-reflex gains and more co-contraction than young adults in all surface conditions.

Balance performance has previously been shown to be poorer in older compared to young adults (Raymakers et al. 2005) and to decrease when standing on a compliant surface (foam) compared to a firm surface (Raymakers et al. 2005). Similarly, our results showed a poorer balance performance, i.e., higher CoM velocities in older than in young adults and when standing on compliant surfaces in both age groups. These findings highlight that age-related impairments and surface compliance both challenge balance control and likely require adaptations in the neural control of balance to maintain stability.

One of the ways in which balance control can be altered with increasing challenge is by down-modulating spinal reflexes. A number of studies have shown down-modulation of the soleus H-reflex with increasing postural instability, such as for instance when decreasing the base of support in standing (Trimble and Koceja 1994), or when comparing walking to standing relaxed (Capaday and Stein 1986) or beam walking to treadmill walking (Llewellyn et al. 1990). Similar down-modulation was found between bipedal and unipedal standing (Pinar et al. 2010; Kim et al. 2013), as replicated in this study. Furthermore, lower H-reflexes in older compared to young adults have been found (deVries et al. 1985; Earles et al. 2000), in line with the age effects in the present study. In unipedal stance on the balance platform, young adults down-modulated the H-reflex gain further with increasing challenge. As lower H-reflexes can be interpreted as a sign of reduced spinal control (Kaplanski 2001), our findings are in line with a shift in balance control from spinal to more supraspinal levels when standing on the more compliant surfaces in young adults, and more supraspinal control overall in older adults. More direct support for a shift from spinal to supraspinal control when standing on unstable surfaces was provided by Solopova et al. (2003) who showed that in adults (aged between 25 and 52 years) TMS-evoked EMG responses of soleus muscle increased, whilst, when controlled for background EMG activity, the H-reflex decreased when standing on an unstable platform compared to a stable platform. However, comparing supported versus unsupported standing, Papegaaij et al. (2016a) found decreased intracortical inhibition but no concurrent changes in H-reflexes.

Interestingly, between unipedal and bipedal stance, both age groups showed down-modulation of the H-reflex. This is in contrast with Koceja et al. (1995) who showed reduced H-reflexes in young, but not in older adults, when decreasing the base of support (prone to standing). However, these authors did find modulation of the H-reflex in a subgroup of older adults with better balance performance (Koceja et al. 1995). The older participants in the present study down-modulated their H-reflexes to some extent and, hence, may have had relatively good balance control. Why they did not further down-modulate H-reflexes in the compliant surface conditions is unknown, but it may simply be because they already had very low reflex amplitudes during unipedal stance on a fixed surface.

While the results presented suggest down-modulation of H-reflexes with increasing task difficulty, an alternative explanation for the decrease in H-reflex gains across stance conditions or surface compliances could be saturation due to increased bEMG. Increasing background activity may lead to a decrease in reflex amplitude as motoneurons that are refractory when the afferent volley arrives will not be recruited. The decrease in H-reflex excitability in unipedal stance compared to bipedal stance could then be the product of this effect, as a tendency towards increased bEMG in unipedal stance was found. Similarly, the down-modulation in young adults with increasing surface compliance could be a result of the increase in bEMG in this group. However, in this case, no changes were found in the absolute H-reflex amplitude, whereas the suggested effect of background activity should also be observable in this parameter. Moreover, the amplitude of the H-reflex is expected to linearly increase with the level of activation up to 60% of maximal activation (Funase and Miles 1999; Taube et al. 2019). Although we have not measured the maximal voluntarily activation of the soleus, excitation higher than 45% of maximal activation on average is not expected in the current tasks (Muehlbauer et al. 2014), which is supported by the normalized EMG data that on average reached up to four times the activity in bipedal standing.

When increasing surface compliance, young adults showed an increase in co-contraction of ankle plantar and dorsi-flexors, while older adults showed higher co-contraction overall compared to young adults. In other studies, increases in co-contraction with increasing task difficulty have been reported for young adults (Selen et al. 2006; Oomen et al. 2015) as well as for older adults (Baudry et al. 2010; Thompson et al. 2018; Acuña et al. 2019). It is well known that increasing co-contraction may enhance control in some conditions (Selen et al. 2005). However, when balancing on a compliant surface, a rigid ankle control induced by co-contraction may limit the flexibility that might be needed on such a surface. On the other hand, it may decrease response times which would benefit control (Oomen et al. 2015). Our results support an adaptive role of muscle co-contraction as we find evidence of increased co-contraction with increasing surface compliance in the young adults, as reported previously (Oomen et al. 2015), but obviously this is not definitive proof of the adaptive nature of this change in control.

It is known that long-term balance training using compliant surfaces leads to improved balance in both young and older adults (Lesinski et al. 2015; Muehlbauer et al. 2015). Our results suggest that such improvements would involve changes in control of the lower leg muscles and findings of decreased H-reflex gains in young adults (Gruber et al. 2007) are in line with this. For older adults, it is unclear what the mechanisms behind such improved balance could be, as we found no changes in H-reflexes and co-contraction with changing surface compliance, and also in long-term training, no changes in H-reflex gains were found in older adults (Ruffieux et al. 2017). Future, long-term studies, in which H-reflexes and co-contraction along with other potential mechanisms of balance improvement are measured could elucidate the how training on compliant surfaces can improve balance control.

### Limitations of the current study

This study has some limitations to be noted. First of all, the number of participants was limited. Next, in the current experimental setup, we could not use a second Optotrak camera array, to ensure uninterrupted collection of coordinates of arm markers. Consequently, we lost some kinematics data due to markers being obscured. For consistency, the arm motion data for all subjects were excluded from the analysis. However, the analysis was redone with arms included for a smaller sample size of subjects (*n*_old_ = 7, *n*_young_ = 8) without missing marker data and very similar results were obtained (as shown in the supplementary material. (1) Another limitation of our study was that the H-reflex is a very sensitive measure, known to be affected by several factors, such as a mental state of the participant, stimulation intensity or the muscle orientation during movement (Tucker et al. 2005; Acuña et al. 2019). The recommended intensity of peripheral nerve stimulation is at 15–25% or 20–40% of Mmax (Crone et al. 1990; Knikou 2008). In line with the other studies (Trimble and Koceja 1994; Lagerquist et al. 2006), we elicited the H-reflex at Hmax, because the recruitment curve for the H-reflex around Hmax is least steep, and thus, any potential changes in electrode location with respect to the nerve (as may occur during balancing) are likely to have less effect. Moreover, Hmax coincided with 15–40% of Mmax for most of the participants. We did not control for movement in our H-reflex analysis. A recent study used a system in which peripheral nerve stimulations were movement triggered during slackline balancing (Giboin et al. 2019), which may increase reliability of outcomes. Finally, we measured H-reflexes of the soleus, not because it has the greatest contribution in mediolateral balance control, but it does have a role in maintaining mediolateral balance (Neptune and McGowan 2016; Cohen et al. 2018) and also the H-reflex in soleus is more reliable than for other ankle muscles (Capaday and Stein 1986). For a further understanding of mediolateral balance control, studying H-reflexes of other lower leg muscles may be needed.

## Conclusion

In conclusion, our study reveals differences in balance control between young and older adults during a unipedal balance task and effects of surface compliance. When faced with a compliant surface, young adults showed a decreased soleus H-reflex gain with a concomitant increase in background EMG activity. Young adults also increased ankle muscle co-contraction with increasing surface compliance. Older adults did not show such modulation in H-reflex and co-contraction.

## Electronic supplementary material

Below is the link to the electronic supplementary material.

Supplementary file1 (DOCX 22 kb)
