# Supplementary material for: Modulation of soleus muscle H-reflexes and ankle muscle co-contraction with surface compliance during unipedal balancing in young and older adults
Source: Exp Brain Res. 2020 Apr 7;238(6):1371–83. doi: 10.1007/s00221-020-05784-0 (PMC7286858; doi:10.1007/s00221-020-05784-0)
Supplement: Supplementary file 1 — Supplementary file1 (DOCX 22 kb) [file 221_2020_5784_MOESM1_ESM.docx]

Supplementary material 1.

In the experiment, arm markers moving forward were blocked from view in a few participants. Therefore, for consistency, all data on arm movement were omitted from analysis. To make sure that this has not affected our conclusions, the analysis has been redone with arms included for those subjects without missing markers for trials with peripheral nerve stimulation *(n_old_ = 8, n_young_ = 10)* and without peripheral nerve stimulation *(n_old_ = 7, n_young_ = 8)*. The effect of surface compliance and age on vCOM (arms included) for the trials without/with peripheral nerve stimulation are mentioned below:

For trials without peripheral nerve stimulation (Surface Compliance, F _(3,39)_ = 4.540, p = 0.008; Age, F _(1,13)_ = 12.206, p = 0.004).

For trial with peripheral nerve stimulation (Surface Compliance, F _(3,48)_ = 7.010, p < 0.001; Age, F _(1,16)_ = 16.758, p < 0.001).

Moreover, results for trials analyzed with and without inclusion of the arms were highly correlated as shown in the figure below.

Fig. 2 Scatter plot of the trials without peripheral nerve stimulation, x-axis vCoM of the whole body, y-axis vCoM of the whole-body excluding arms
